# Thermal plasticity in farmed, wild and hybrid Atlantic salmon during early development: has domestication caused divergence in low temperature tolerance?

**DOI:** 10.1186/s12862-016-0607-2

**Published:** 2016-02-16

**Authors:** Monica Favnebøe Solberg, Lise Dyrhovden, Ivar Helge Matre, Kevin Alan Glover

**Affiliations:** Population Genetics Research Group, Institute of Marine Research, Bergen, Norway; Matre Research Station, Institute of Marine Research, Matredal, Norway; Department of Biology, Sea Lice Research Centre, University of Bergen, Bergen, Norway

**Keywords:** Ecology, Escapees, Growth, Introgression, Local adaptation, Reaction norms, Salmon strains, Survival, Phenotypic plasticity, Salmonids

## Abstract

**Background:**

In the past three decades, millions of domesticated Atlantic salmon *Salmo salar* L. have escaped from farms into the wild. Their offspring display reduced survival in the natural environment, which demonstrates that gene-flow is likely to have a negative effect on wild populations. However, inter-population differences in introgression of farmed salmon have been observed, and the underlying ecological mechanisms remain enigmatic. We hypothesised that domestication-driven divergence in tolerance to low temperatures during early development may contribute to lower survival of farmed salmon offspring in the wild, which in turn, may influence patterns of introgression among populations exposed to different temperature regimes. We reared the offspring of 35 families of wild, farmed and hybrid origin at three temperatures (3.9, 5.6 and 12 °C) from the onset of exogenous feeding and throughout their first summer. Thermal reaction norms for growth and survival were investigated along the gradient.

**Results:**

The main results of this study, which is based upon the analysis of juvenile salmon from five wild strains, two farmed strains and two hybrid strains, can be summarised as; (i) salmon of all origins were able to successfully initiate feeding at all temperatures and similar survival reaction norms were detected in all strains across the temperature gradient; (ii) deviating growth reaction norms were detected between strains, although this result was most likely due to an overall lack of growth in the lower temperature treatments.

**Conclusions:**

This study revealed no evidence of domesticated-driven divergence in low temperature tolerance in Atlantic salmon during early development. Although the potential interaction between low temperature and other river-specific factors cannot be excluded, our results indicate that the reduced survival of farmed offspring in the wild is not explained by farmed salmon displaying impaired abilities to initiate feeding at low temperatures. We therefore suggest that the observed inter-population patterns of introgression are not low-temperature driven and that other ecological or biological factors may explain why detection of farmed salmon in wild rivers is not synonymous with introgression. In general, our results support the literature indicating that phenotypic plasticity instead of thermal adaption has been selected for in Atlantic salmon.

**Electronic supplementary material:**

The online version of this article (doi:10.1186/s12862-016-0607-2) contains supplementary material, which is available to authorized users.

## Background

All salmonid fishes display a thermal range within which they can either tolerate or optimally perform [1, 2]. For the Atlantic salmon (*Salmo salar* L*.*), an anadromous species inhabiting cold-water rivers in the northern hemisphere, thermal limitations for freshwater growth have been estimated to be between 1.0–7.7 and 23.3–26.7 °C for the lower and upper thresholds respectively [3–6]. For thermal tolerances in freshwater, these ranges are typically wider than those for growth [7, 8].

Phenotypic plasticity plays a significant role in thermal tolerance and thermal growth optimums, and strong positive effects of acclimatisation have been observed [6, 9]. Furthermore, it has been argued that there is limited evidence of thermal adaptation at the population level, with the possible exception of adaptations to very cold conditions [1], or specifically in terms of growth efficiency [2]. Nevertheless, the possibility that adaptive divergence for both temperature tolerance and growth optimums may be displayed among wild Atlantic salmon populations inhabiting streams with vastly different thermal regimes has been discussed [10, 11].

In connection with commercial aquaculture, the Atlantic salmon has been subject to domestication selection since the 1970’s [12]. At present, Norwegian breeding programs, that are the most advanced globally for this species, have exceeded 10 generations [13, 14]. Breeding initially targeted growth that typically displays high heritability estimates [15], but thereafter included other traits such as flesh and carcass characteristics, delayed maturation and disease resistance [12]. The results of these selection programs have been documented, with growth rates that sometimes exceed double the growth rates of wild salmon under identical hatchery conditions [16–18]. However, due to mechanisms such as inadvertent co-selection, relaxed selection and trade-offs [19], these genetic changes have been at the expense of other characteristics, such as stress resistance [18, 20], aggression [16, 21], and anti-predator behaviour [16, 22]. Studies also show that the offspring of farmed salmon display reduced survival in the wild when compared to the offspring of wild salmon [23–25]. Documented reduced predator awareness [16, 22] may explain some of the survival differences between the offspring of farmed and wild salmon in the natural environment, even though increased predation susceptibility in farmed salmon is yet to be documented [26, 27]. Furthermore, population density may shift the competitive balance between farmed and wild salmon in nature [28, 29], while other potential mechanisms, such as deviating responses to ambient environmental conditions, may also contribute to the lower survival of farmed Atlantic salmon offspring in the wild.

Farmed Atlantic salmon may experience various temperatures during production. However, during the transition phase from alevin to fry, which is referred to as “start-feeding”, water temperatures are often raised to 10 °C or warmer [30]. This is in order to speed up this production phase where the fish are both resource demanding to handle as well as sensitive to environmental quality. Thus, in this early life-history phase, domesticated Atlantic salmon have had a reduced exposure to low and potentially critically low water temperatures. Based upon the documentation of thermal adaptation in other fish species [31–34], as well as the rapid evolution of this trait [31, 32], it is possible that farmed salmon may have adapted to the heated domestic environment, and as a consequence, exhibit reduced tolerance to cold temperatures, especially during early developmental stages. In the wild, emergence and initiation of feeding is a period of high natural mortality where density dependent and independent mechanisms are at work [35, 36]. In many rivers, the alevin to fry transition may occur at the lower end of the temperature limitation for growth in this species. Thus, where farmed escaped salmon have interbred with wild populations in cold rivers, or in years where the following spring is cold, their offspring may be at an extra competitive disadvantage to the offspring of wild salmon. Investigating these potential mechanisms of divergence in survival between farmed and wild salmon in the wild is of importance in conservation biology, as farmed Atlantic salmon have interbred to varying degrees in a number of wild populations [29, 37, 38].

The present experiment was designed to address the following question; do farmed salmon display reduced abilities to initiate start-feeding at very low temperatures in comparison to wild salmon? We start-fed and reared a total of 35 full and half-sibling families originating from two farmed strains and five wild populations in a common-garden experiment design at three different temperatures. Mortality was recorded daily and the survival reaction norm along the critically low temperature gradient was investigated. In addition, the thermal reaction norm for growth was investigated in a random subset of surviving individuals.

## Methods

### Experimental crosses and rearing

**Fig. 1:**
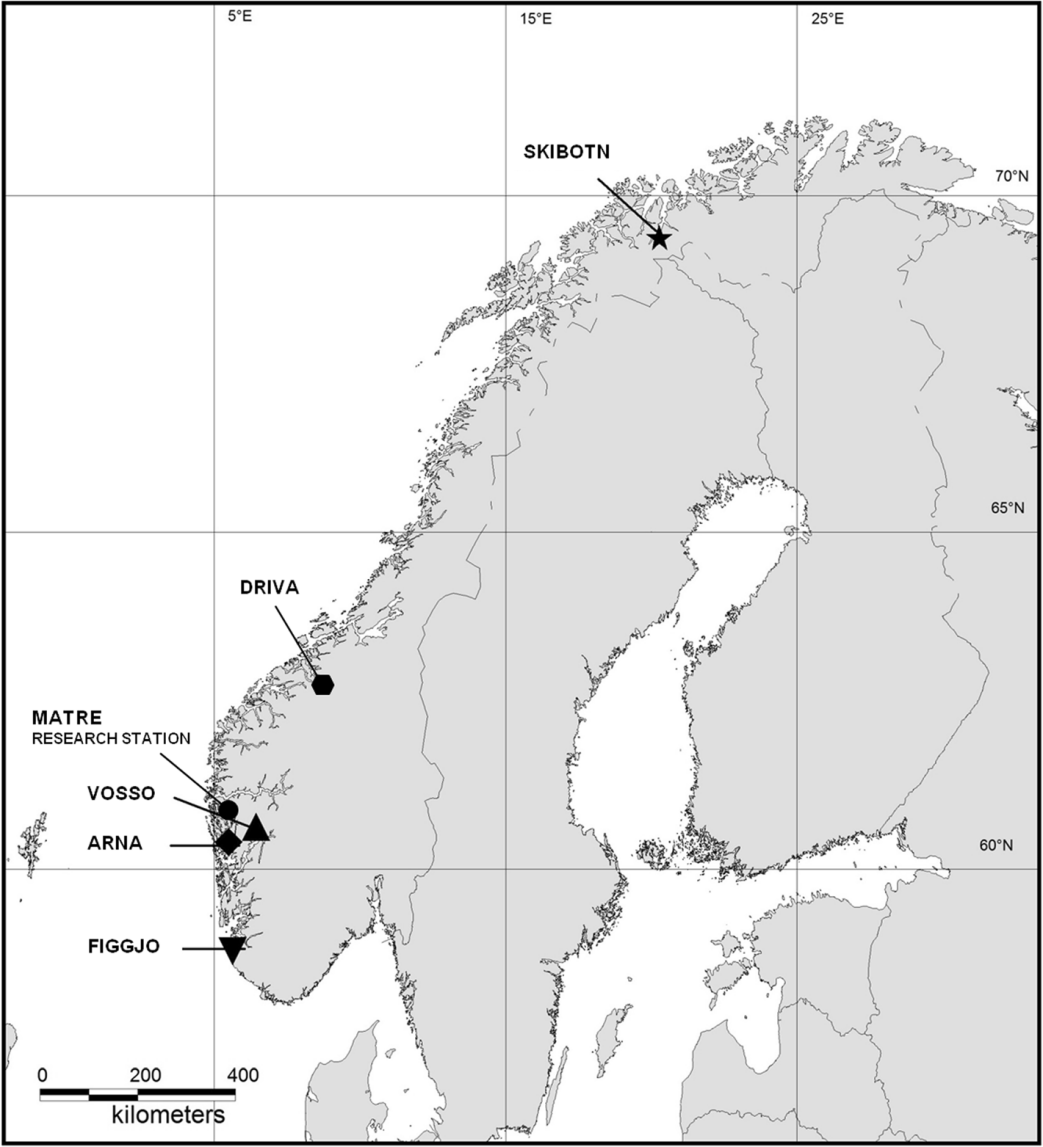
Map showing the location of the rivers of the five Norwegian wild salmon populations included in this study: Figgjo, Arna, Vosso, Driva and Skibotn. The Matre research station is also marked where the fish were reared throughout the experimental period. Parental Atlantic salmon of the Figgjo, Arna and Vosso strain were collected from their respective rivers, while parental salmon of the Driva and Skibotn strains were reared in the gene bank at Haukvik, central Norway

**Fig. 2:**
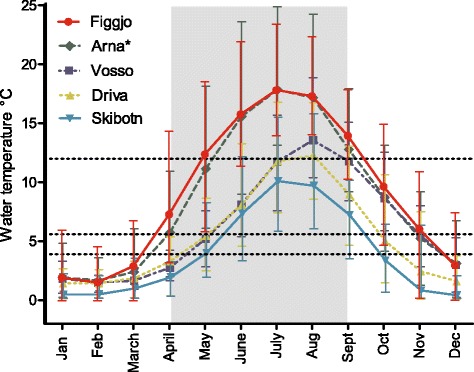
Average monthly water temperatures for the five rivers in the period 2002–2012. Error bars show the minimum and maximum temperatures registered. Shaded area illustrates the time period of the study, while dotted lines illustrates the three experimental temperature treatments. For Skibotn, only data from 1980–1986 were available. * As data from River Arna was not available, temperature data from the nearby river Os (60 °18’N, 5°47’E) was used to create this figure. The River Os and River Arna are located within the same watershed, receive water from the same mountain and their temperature regimes are expected to be similar

**Fig. 3:**
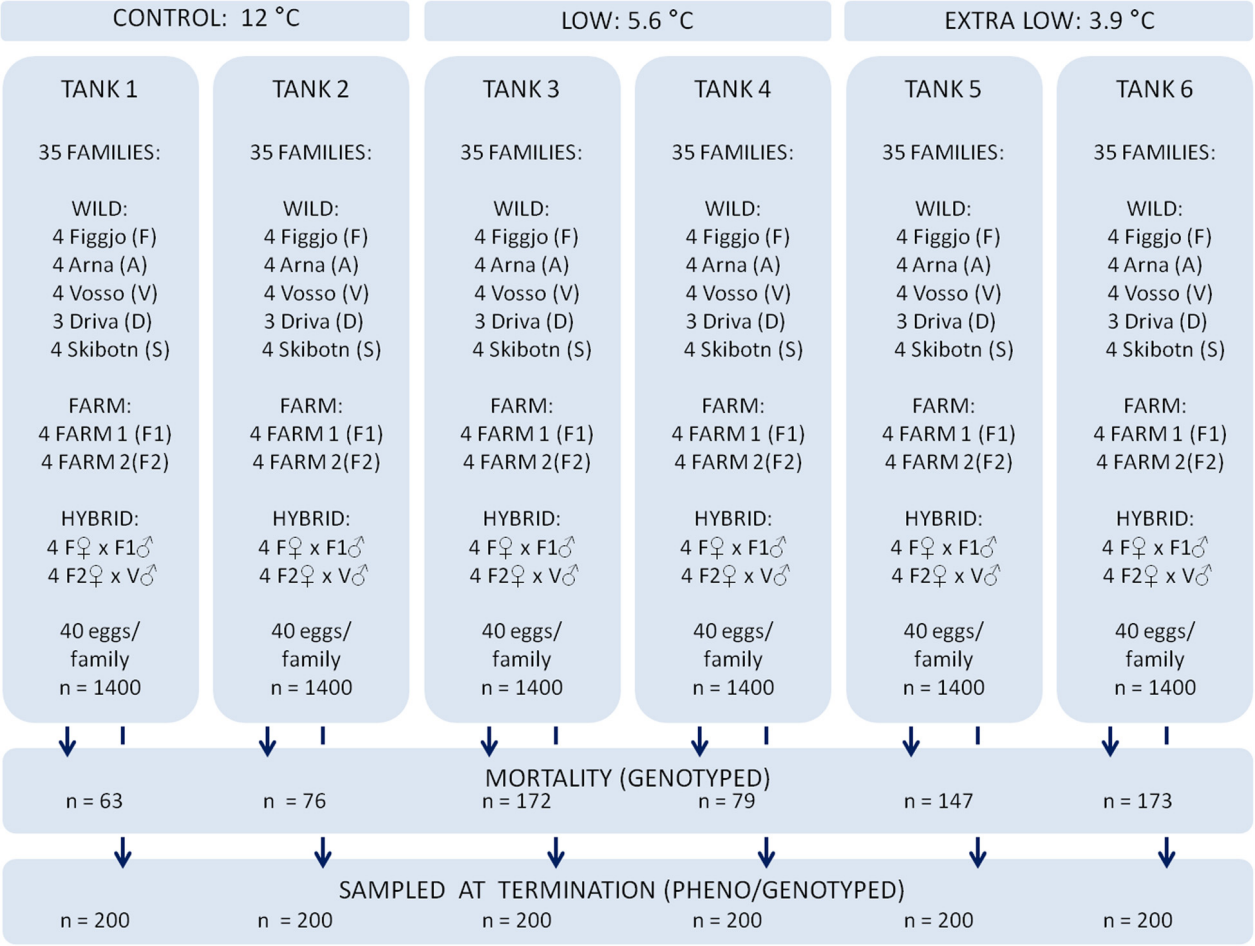
Experimental design. A total of 8400 Atlantic salmon of farmed, hybrid and wild origin were included in this study. Experimental replicates were sorted out at the eyed-egg stage. The temperature treatments were initiated at the onset of exogenous feeding, while the experiment was terminated after the first summer. All individuals that died during the experimental period were DNA sampled and assigned to family of origin in order to investigate the strains thermal reaction norm for survival. At termination of the experiment a random subset of the surviving individuals were sampled in order to investigate the strains thermal reaction norm for growth

Atlantic salmon were in November 2012 sampled from five wild populations representing a wide variation in river temperature regimes (Figs. 1 and 2) and from two commercial farmed strains and used to generate nine experimental strains for this study: five pure wild strains, two pure farmed strains and two wild/farmed F1 hybrid strains. A total of 35 families were included in the study, using four families per strain with the exception of one wild strain where only three families were included (Fig. 3).

Wild parental salmon were captured in the rivers Figgjo and Arna and temporarily transferred to the local hatcheries. Later, either live fish or gametes were transport to the Matre research station where the experiment was conducted. Arna salmon were stripped on November 12, 2012, while Figgjo salmon were stripped on November 14, 2012. One of the fish collected in the river Figgjo had a tag which demonstrated that it originated from the nearby river Ims.

The population of the river Vosso has since the early 1990’s been conserved by the Norwegian Gene Bank for wild Atlantic salmon, due to a severe decline in the population and an increase in farmed escapees at the spawning ground. In the gene bank, maintenance of wild salmon and their offspring are performed without any form of directional selection. The adult Vosso salmon used as parents in the present study had been reared in the hatchery until the smolt stage before being released into the sea. Returning fish were caught in the river by angling or nets, and held in the Voss hatchery until stripping. On November 15, 2012, gametes were collected from adults in the Voss hatchery, transported to the Matre research station, and fertilised on the same day.

The rivers Driva and Skibotn have been conserved by the Norwegian Gene Bank for Atlantic salmon, in the same manner as the Vosso strain, due to repeated infestation of the parasitic monogenean *Gyrodactylus salari*. Gametes were collected from spawners reared in freshwater in the gene bank at Haukvik, central Norway, for one to three generations, and shipped overnight to the Matre research station. Approximately 24 h post stripping, Skibotn gametes were fertilised on November 13, 2012, while Driva gametes were fertilised on November 20, 2012.

The SalmoBreed farmed strain is based upon genetic material from several Norwegian farmed strains that have been under commercial selection since the late 1960’s and early 1970’s, and was commercially established in 1999. The commercial Mowi strain from Marine Harvest is the oldest Norwegian farmed strain. Large multi-sea winter fish collected from the River Bolstad in the Vosso watercourse and the River Åroy, in addition to wild salmon caught in the sea outside of western Norway, near Osterfjord and Sotra were used to establish this strain in 1969. Brood fish of the approximately 10^th^ generation of selected parents from the SalmoBreed and Mowi strains were used here. Gametes were collected at the SalmoBreed breeding station at Osterøy, and at the Mowi breeding station at Askøy, both in western Norway, and thereafter transported to Matre. All gametes were fertilised on 14–15 November, 2012.

The two F1 hybrid strains were generated by crossing females of one of the farmed strains with males of the wild Figgjo strain, while males of the other farmed strain were crossed with females of the wild Vosso strain. The hybrid strains were established on November 14–15, 2012. Both farmed strains will from here-on referred to as Farm1 and Farm2 (random order).

Adipose fin clips were collected from all parental salmon for parentage assignment of offspring upon termination of the experiment. Scale samples were also collected from all wild brood stock. This was done in order to ensure that all brood stock were of wild origin, and not farmed escapees [39].

### Experimental conditions

All families were established in the period November 12–20, 2012, at the Matre research station. At the eyed-egg stage, mean family egg diameter was recorded and 40 eggs per family were sorted into six replicates. The experiment was initiated at the time of start-feeding, on April 2, 2013, in 1 m diameter tanks with 3.9 °C, 5.6 and 12 °C water (two replicates per temperature) (Fig. 3). These treatments will from here-on be referred to as the extra low, low and control temperature treatments, respectively.

The fish were reared under standard hatchery conditions with a 24 h light regime. A commercial pelleted diet was provided *al libitum.* To control for an increase in biomass during the course of the experiment, both control treatment replicates were split into two tanks on July 16, 2013. Thus upon termination, the control treatment consisted of four tanks, while the low and extra low temperature treatments both consisted of two tanks each. Temperature regimes were remained throughout the experimental period totalling 21 weeks.

### Ethics statement

The experiment was performed in accordance with the general guidelines for animal studies, the Animal Research Reporting In Vivo Experiments (ARRIVE) guidelines [40]. All salmon were reared under standard commercial conditions at temperature regimes within the natural range observed in nature. By The Norwegian Regulation on Animal Experimentation, such conditions do not fall under the category of animal experiments where approval of the experimental protocol by the Norwegian Animal Research Authority (NARA) is needed. However, welfare and use of experimental animals was performed in strict accordance with the Norwegian Animal Welfare Act. In addition, all personnel involved in the experiment had undergone training approved by the Norwegian Food Safety Authority, which is mandatory for all personnel running experiments involving animals included in the Animal Welfare Act.

### Sampling, genotyping and parentage testing

Dead fish were sampled from all tanks daily and stored in ethanol. The experiment was terminated on August 28, 2013. At this stage, 200 individuals were randomly sampled from all six replicates. Sampled individuals were euthanised with metacain (Finquel Vet, ScanVacc, Årnes, Norway), wet weight and fork length measured. In addition a fin tissue sample was collected for DNA parental assignment.

DNA was isolated from parental and offspring tissue samples. Tissue samples and six polymorphic microsatellite loci were genotyped on an ABI3730XL sequencer. Genotypes were identified using GeneMapper V4.0., and offspring assigned to family by the use of FAP v3.6 [41]. More extensive details with respect to the exact genotyping procedure are available elsewhere [42].

### Statistical analysis

#### Mortality

All statistical analyses were performed in R (v 3.1.0.) [43], with critical P-values set to 0.05.

In order to investigate if overall mortality varied between the three temperature treatments, between the nine experimental strains, or was influenced by egg size, a generalized linear mixed effect model (GLMM) was fitted using the *glmer* function in the lme4 package [44]. The full model tested for the effect of treatment (T), strain (S) and the continuous effect of mean family egg diameter (E), as well as all the interactions between treatment and strain (TS) and between treatment and egg size (TE), upon mortality (M). The interaction between strain and mean family egg size was not included in the full model due to the few observations of egg size within strains. Different variance patterns across treatment replicates was investigated and controlled for by including replicate nested within treatment (r(T)) as a random intercept factor, while differences in variance patterns between families across treatments was investigated by allowing the random effect of family nested within strain (f(S)), i.e., random intercept, vary across treatments (T), i.e., random slope:

1 $$ \mathrm{logit}(M)=\alpha +{\beta}_1T+{\beta}_2S+{\beta}_3E+{\beta}_4TS+{\beta}_5TE+{b}_{r(T)}+{b}_{f(S)}T+\varepsilon $$

where *α* is the intercept and *ε* is a random error. Due to survival being binary data the binomial distribution was selected with a logistic link function and the model was fitted using the Laplace approximation. To achieve a better convergence of the model, the egg size was centred (overall mean egg size subtracted from all observations). The significance level of the random intercept effects was assessed by fitting the full fixed model while only including one random intercept effect at a time, before plotting the 95 % prediction intervals of the random effect, using the *dotplot* function of the lattice package [45]. If all of the prediction intervals of the random intercept effects overlapped zero, they were considered significant. Further selection on the random family effect structure, i.e., a random intercept model versus a random slope and intercept model, was performed by backward selection. For that reason a likelihood ratio test (LRT) was performed on a full fixed effect model fitted with the two random effect structures (Additional file 1: Table A1). The fixed effects structure was identified by backward model selection, based upon AIC values [46], while using the *drop1* function (Additional file 1: Table A2). Insignificant variables were removed from the model, interaction terms before the variables themselves, until no further improvement of the model fit were detected:

2 $$ \mathrm{logit}(M)=\alpha +{\beta}_1T+{\beta}_2S+{\beta}_3E+{b}_{r(T)}+{b}_{f(S)}+\varepsilon $$

Pair-wise comparison between treatments and strains (Additional file 1: Table A3) were performed using the *testinteractions* function in the phia package [47], which uses an Holm’s adjustment of the P-values. Estimated traits means were retrieved by the use of the *interactionMeans* function in the same package.

#### Growth

In order to investigate the influence of temperature treatment, strain and egg size upon body weight at termination, a linear mixed effects (LME) model was fitted using the *lmer* function in the lme4 package [44]. We tested for the categorical effects of temperature treatment (T) and strain (S) and the continuous effect of mean log_10_ family egg diameter (E), as well as the interactions between treatment and strain (TS) and treatment and egg size (TE), upon log_10_ body weight (BW). The interaction between strain and egg size was not included in the full model for the same reason as stated above. Different variance across tanks (8) and/or treatment replicates (6) was investigated and controlled for by including tank nested within replicate and treatment (t(r(T)) as a random intercept factors, while differences in variance patterns between families across treatments was controlled for by allowing the random effect of family nested within strain (f(S)), i.e., random intercept, vary across treatments (T), i.e., random slope:

3 $$ BW=\alpha +{\beta}_1T+{\beta}_2S+{\beta}_3E+{\beta}_4TS+{\beta}_5TE+{b}_{t\left(r(T)\right)}+{b}_{f(S)}T+\varepsilon $$

where *α* is the intercept and *ε* is a random error. Model selection was performed backwards by the use of the *step* function in the lmerTest package [48]. By this procedure, insignificant random effects were eliminated, followed by the removal of insignificant fixed effects (Additional file 1: Table A4). Interaction terms were removed before the variables themselves:

4 $$ BW=\alpha +{\beta}_1T+{\beta}_2S+{\beta}_3E+{\beta}_4TS+{b}_{t\left(r(T)\right)}+{b}_{f(S)}T+\varepsilon $$

P-values for the random effects were calculated based upon likelihood ratio tests, while F-statistics, denominator degrees of freedom and P-values calculated based on Satterthwaite's approximations were presented for the fixed effects [48]. For the significant categorical fixed effects, least squares means and differences of least squares means were calculated, i.e., pair-wise parameter level tests (Additional file 1: Table A5-8). Estimated trait means were retrieved from the model output, and the estimated mean for log10 body weight was thereafter back transformed.

## Results

### Genotyping and parentage testing

In total, 710 salmon died during the experiment. Of these, 701 individuals were successfully assigned to family. The nine individuals that could not be assigned to family were excluded from the survival analysis, and survival rate was calculated as number of individuals at start minus number of dead individuals assigned to each respective family.

Of the 1200 individuals sampled at the end of the experiment, 1199 were successfully identified to family. In addition, four individuals were excluded from the growth analyses due to sampling errors.

### Influence of temperature treatment on survival

**Fig. 4:**
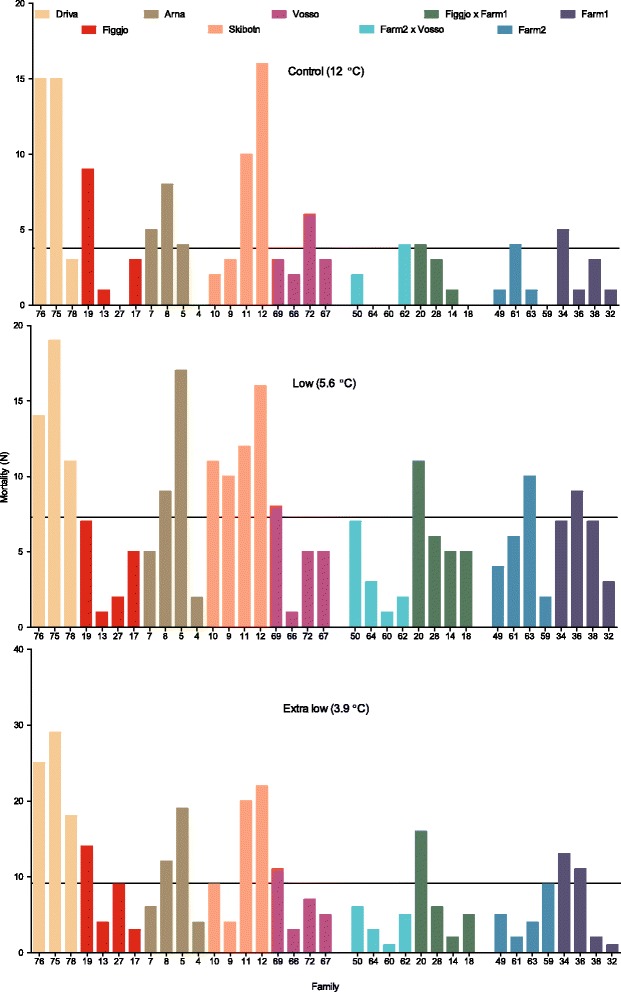
Family mortality. The observed number of the initial 80 individuals from each of the 35 Atlantic salmon families, in each temperature treatment, that died during the experimental period from start-feeding to after the first summer. Families within strains are ranked by the average weight of their surviving siblings in the control treatment (increasing order). Lines illustrate the average number of individuals that died per family, in each treatment. Survival was significantly lower in the extra low temperature treatment (89 %), as compared to the low (91 %) and control (95 %) temperature treatment

**Fig. 5:**
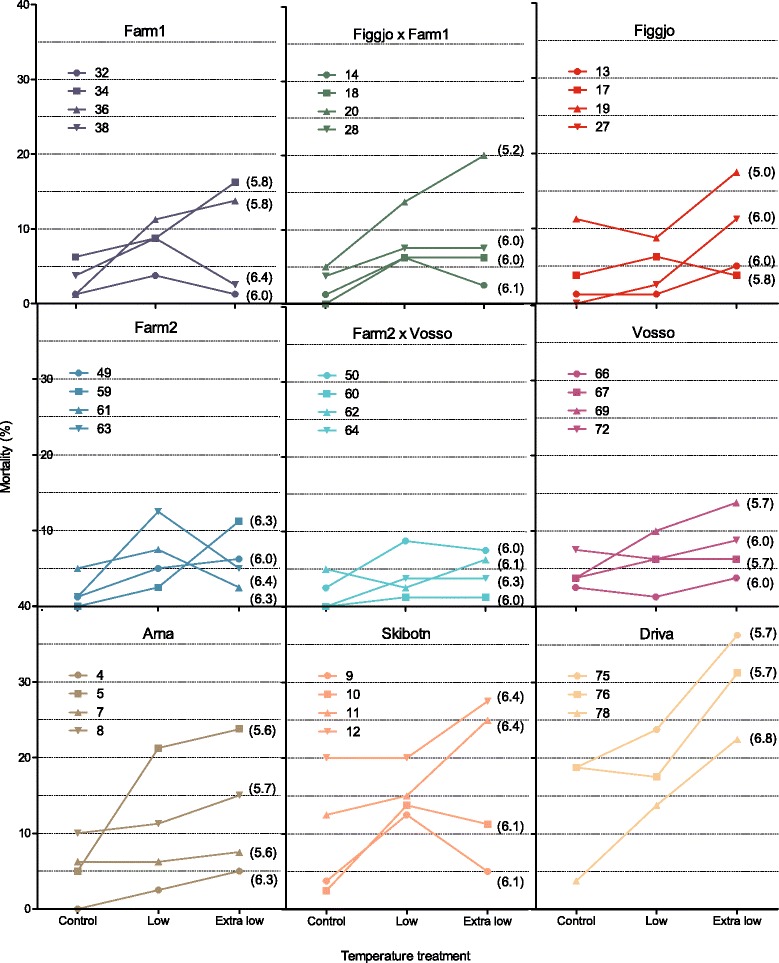
Survival reaction norm. Observed survival (%) reaction norms along the thermal gradient, for all 35 families included in this study. All nine strains displayed a similar survival reaction norm, hence Atlantic salmon of farmed, hybrid and wild salmon managed to initiate start-feeding at cold temperatures in a similar manner. Two strains, Driva and Skibotn, distinguished themselves by displaying the lowest survival rate in all treatments. Numbers in brackets illustrates the family’s mean egg diameter (mm)

Observed survival in the extra low, low and control temperature treatments were 89, 91 and 95 %, respectively (Figs. 4 and 5), and thus significantly higher in the control treatment than in the extra low temperature treatment (Table 1, Additional file 1). An overall positive effect of egg size, in addition to differences in variance patterns across replicates and families, was detected and therefore controlled for in the generalized linear mixed effect model (Additional file 1).

**Table 1:** Pair-wise comparisons of estimated survival between strains, and relative survival of strains within each treatment

		Farm1 (F1)			Farm2 (F2)			Figgjo x Farm1			Farm2 x Vosso			Figgjo (F)			Vosso (V)			Arna (A)			Driva (D)			Skibotn (S)		
Temp (° C)		12	5.6	3.9	12	5.6	3.9	12	5.6	3.9	12	5.6	3.9	12	5.6	3.9	12	5.6	3.9	12	5.6	3.9	12	5.6	3.9	12	5.6	3.9
Est surv. (%)		96.5	93.8	91.5	96.5	93.8	91.5	97.2	94.9	93.0	98.0	96.4	95.0	97.8	96.0	94.5	97.2	95.0	93.1	96.1	93.1	90.6	86.8	78.2	72.0	89.7	82.7	77.3
F1		-	-	-																			**			*		
F2	1:	1.00	1.00	1.00	-	-	-																**			*		
F x F1	1:	0.99	0.99	0.98	0.99	0.99	0.98	-	-	-													***			**		
F2 x V	1:	0.98	0.97	0.96	0.98	0.97	0.96	0.99	0.98	0.98	-	-	-										***			***		
F	1:	0.99	0.98	0.97	0.99	0.98	0.97	0.99	0.99	0.98	1.00	1.00	1.01	-	-	-							***			***		
V	1:	0.99	0.99	0.98	0.99	0.99	0.98	1.00	1.00	1.00	1.01	1.02	1.02	1.01	1.01	1.02	-	-	-				***			**		
A	1:	1.00	1.01	1.01	1.00	1.01	1.01	1.01	1.02	1.03	1.02	1.04	1.05	1.02	1.03	1.04	1.01	1.02	1.03	-	-	-	**					
D	1:	**1.11**	**1.20**	**1.27**	**1.11**	**1.20**	**1.27**	**1.12**	**1.21**	**1.29**	**1.13**	**1.23**	**1.32**	**1.13**	**1.23**	**1.31**	**1.12**	**1.21**	**1.29**	**1.11**	**1.19**	**1.26**	-	-	-			
S	1:	**1.08**	**1.13**	**1.18**	**1.08**	**1.13**	**1.18**	**1.08**	**1.15**	**1.20**	**1.09**	**1.17**	**1.23**	**1.09**	**1.16**	**1.22**	**1.08**	**1.15**	**1.20**	1.07	1.13	1.17	0.97	0.95	0.93	-	-	-

Upper triangular illustrates the *P*-values, * = *P* < 0.05, ** = *P* < 0.01, *** = *P* < 0.001, of the pair-wise comparisons of estimated survival between strains, as retrieved from the output of the generalized linear mixed effect model 1 (Additional file 1). Lower triangular shows the relative difference, i.e., ratio, in estimated survival of strains within each treatment. Significant overall differences in survival between strains are marked in bold

### Influence of strain on survival

Strains that survived well in the control treatment also survived well in the low and extra low temperature treatments, while strains that survived poorly in the control treatment also survived poorly in the low and extra low temperature treatment. Thus, no interaction was detected between treatment and strain upon survival (Figs. 4 and 5 and Additional file 1). Two strains, Driva and Skibotn, distinguished themselves by displaying the lowest survival rate in all treatments (Table 1).

### Influence of temperature treatment on body weight

**Fig. 6:**
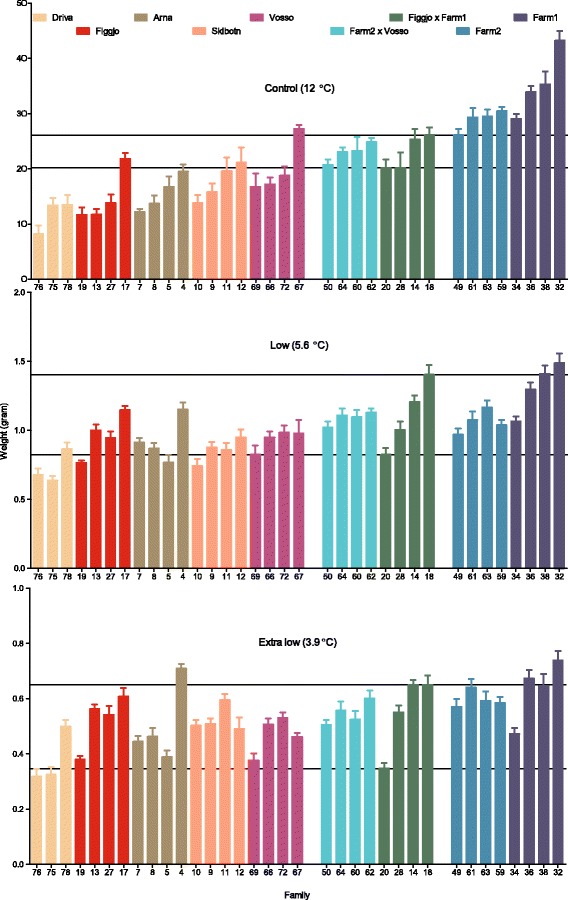
Mean family weights. Observed mean family weight (g) of all 35 Atlantic salmon families, in all temperature treatments, at the termination of the experiment after the first summer. Growth was significantly higher in the control treatment, as compared to the low and extra low temperature treatment. More overlap in growth between the farmed, hybrid and wild families were detected in the low and extra low treatment, than in the control treatment. Lines illustrate the best and worst growing hybrid family, in each treatment. Error bars show the standard error. Families within strains are ranked by their average weight in the control treatment (increasing order)

**Fig. 7:**
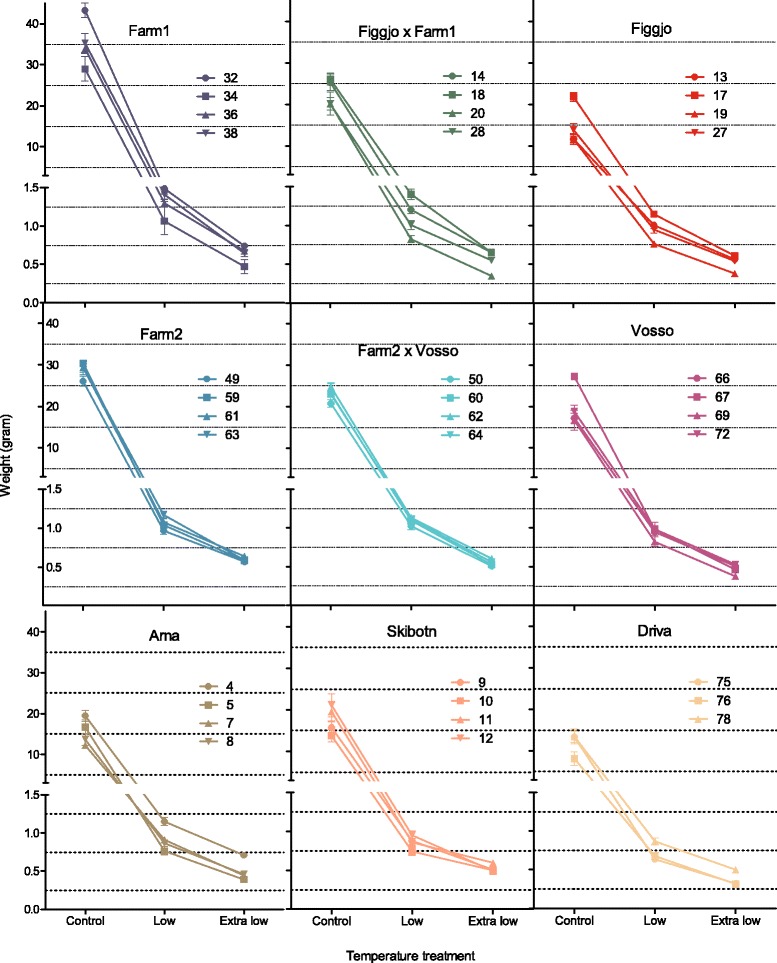
Growth reaction norm. Observed growth reaction norms along the thermal gradient, for all 35 families included in this study. Growth rates decreased along the temperature gradient, and few differences in growth between the nine Atlantic salmon strains were detected at the low and extra low temperature treatments, in contrast to at the control treatment. This resulted in deviating thermal reaction norms for growth in the strains investigated here. Error bars show the standard error

Fish size was significantly higher in the control treatment, than in the low and extra low temperature treatments (Figs. 6 and 7 and Additional file 1). Observed average weights were 22.2 g in the control treatment, 1.0 g in the low temperature treatment and 0.53 g in the extra low temperature treatment. Hence fish in the low and extra low treatment were out-grown by fish in the control treatment by a ratio of 1:22, and 1:42, respectively. Growth in the two cold temperature treatments was also significantly different (Additional file 1), and fish in the extra low treatment were out-grown by fish in the low treatment by 1:1.9.

A significant positive effect of egg size on fish weight was detected (Additional file 1), in addition to differences in variance patterns across tanks and/or replicates, and families across treatments. This was controlled for in the final linear mixed effect model (Additional file 1).

### Influence of strain on body weight

Fewer differences were detected between the strains at the cold temperature treatments, as compared to in the control treatment (Figs. 6 and 7). Thus, the relative difference in weight between strains varied between the treatments (Table 2), and a significant interaction between treatment and strain was detected (Additional file 1).

**Table 2:** Pair-wise comparisons of estimated body weight between strains within each treatment, and their relative weight

		Farm1 (F1)			Farm2 (F2)			Figgjo x Farm1			Farm2 x Vosso			Figgjo (F)			Vosso (V)			Arna (A)			Driva (D)			Skibotn (S)		
Temp (° C)		12	5.6	3.9	12	5.6	3.9	12	5.6	3.9	12	5.6	3.9	12	5.6	3.9	12	5.6	3.9	12	5.6	3.9	12	5.6	3.9	12	5.6	3.9
Est. W (g)		34.2	1.27	0.62	27.5	0.97	0.52	22.4	1.14	0.58	21.9	1.05	0.52	14.2	1.05	0.59	19.3	0.95	0.50	15.3	0.99	0.54	10.6	0.69	0.36	15.1	0.76	0.45
F1		-	-	-		**		**			**	*		***	*		***	***		***	**		***	***	***	***	***	*
F2	1:	1.24	**1.31**	1.18	-	-	-							***			*			***			***	***	*	***	*	
F x F1	1:	**1.52**	1.12	1.06	1.23	0.85	0.90	-	-	-				**				*		**			***	***	**	**	***	
F2 x V	1:	**1.56**	**1.21**	1.19	1.26	0.92	1.01	1.03	1.08	1.12	-	-	-	**						*			***	***	*	**	***	
F	1:	**2.40**	**1.21**	1.05	**1.93**	0.92	0.89	**1.58**	1.08	1.00	**1.54**	1.00	0.89	-	-	-	*							***	**		***	
V	1:	**1.77**	**1.35**	1.24	**1.43**	1.03	1.05	1.17	**1.20**	1.17	1.14	1.11	1.04	**0.74**	1.11	1.17	-	-	-				***	**	*		*	
A	1:	**2.24**	**1.28**	1.15	**1.80**	0.98	0.97	**1.47**	1.14	1.08	**1.43**	1.06	0.96	0.93	1.06	1.09	1.26	0.95	0.93	-	-	-	*	***	*		**	
D	1:	**3.23**	**1.85**	**1.71**	**2.60**	**1.41**	**1.45**	**2.12**	**1.65**	**1.61**	**2.07**	**1.53**	**1.44**	1.35	**1.53**	**1.62**	**1.82**	**1.37**	**1.38**	**1.45**	**1.44**	**1.49**	-	-	-	*		
S	1:	**2.27**	**1.67**	**1.36**	**1.83**	**1.27**	1.16	**1.49**	**1.49**	1.29	**1.45**	**1.38**	1.15	0.94	**1.38**	1.29	1.28	**1.24**	1.10	1.01	**1.30**	1.19	**0.70**	0.90	0.80	-	-	-

Upper triangular illustrates the *P*-values, * = *P* < 0.05, ** = *P* < 0.01, *** = *P* < 0.001, of the pair-wise comparisons of estimated weight of strains within each temperature treatments, i.e., differences in least squares means, as retrieved from the output of the linear mixed effect model 2 (Additional file 1). Lower triangular shows the relative difference, i.e., ratio, in estimated weight of strains within each treatment. Significant results are marked in bold

In the control treatment, the two farmed strains were larger than the two hybrid strains, which again were larger than the five wild strains (Table 2). Although not all pair-wise comparisons between strains of farmed, hybrid and wild origin were significant (Table 2, Additional file 1), no overlap at the strain level was detected between the three genetic origins (Figs. 6 and 7). In the low temperature treatment, some overlap at the strain level was detected between farmed, hybrid and wild salmon. However, out of the four best growing strains, three were of farmed or hybrid origin, while four of the five worst growing strains were of wild origin. In the extra low treatment, two of the four best growing strains were of farmed or hybrid origin, while three of the five worst growing strains were of wild origin. In all treatments, Farm1 displayed the highest, while Driva displayed the lowest growth.

## Discussion

In this study, thermal plasticity of Atlantic salmon of farmed, wild and F1 hybrid origin was investigated by describing their reaction norm for survival and growth across a temperature gradient ranging from 3.9 to 12 °C. The overall results of this study indicate that phenotypic plasticity in temperature tolerance, instead of adaptation to ambient temperatures [49], have been selected for in Atlantic salmon. Although strains displayed overall differences in survival, their thermal reaction norms were similar, and salmon of all genetic backgrounds were able to successfully initiate feeding at all temperatures. Although the thermal reaction norms for growth differed between the strains, this result was likely influenced by the fact that in general, few differences were detected among the strains in the cold temperature treatments due to overall lack of growth in those treatments.

### Thermal adaption

Within salmonids, population-specific thermal adaption has been debated [10, 11], and the potential of local adaptation towards diverging thermal regimes have been indicated to be low, and if present, populations inhabiting extreme environments should be most likely to reveal this feature [50]. Two potential hypothesis have been suggested towards thermal adaption; the local thermal optima adaptation hypothesis [51], and the countergradient hypothesis [52, 53].

#### The local thermal optima adaptation hypothesis

The local optima hypothesis predicts that a population’s optimal temperature for growth would be linked to the temperature in the local river. Thus, populations experiencing warm ambient temperatures should perform better at such temperatures, and *vice versa* for populations experiencing cold ambient temperatures. Farmed salmon have been under the process of directional selection and domestication for approximately 40 years, and as a result been primarily exposed to temperatures above 10 °C during early development. However, in comparison with wild salmon, farmed salmon did not display reduced survival, i.e., reduced abilities to initiate feeding, at cold temperatures in this study. Nevertheless, smaller differences in growth between salmon of farmed and wild origin were detected in the cold temperatures. While this could indicate that the relative growth of wild salmon in comparison with the farmed salmon is greater at the lower temperatures, i.e., farmed salmon out-grows wild salmon to a lesser degree as temperature decreases, it is most likely that this result is confounded by the fact that growth in general was very low at these cold temperatures and therefore the farmed salmon had limited opportunity to out-grow the wild salmon. If the intrinsic growth rate of salmon of farmed and wild origin display parallel growth trajectories across thermal time, then a similar difference in growth between strains may have been observed at all temperatures if treatments were terminated at the same number of degree days, instead of at the same number of calendar days. Growth of farmed and wild Norwegian salmon have previously been demonstrated to be similar up until the onset of exogenous feeding [54], which indicates that differences in growth will be hard to detect at early life-history stages, as only limited growth has occurred since feeding was initiated at these cold temperatures.

All wild strains investigated here originated from rivers known to display temperatures below the coldest temperature treatment used in the present study, at least during parts of the year (Fig. 2). Thus, detection of similar survival reaction norms among the wild populations was not unexpected. However, populations inhabiting the rivers Figgjo and Arna are the most likely to experience temperatures resembling the low temperature treatment during early development, while the populations in the rivers Vosso, Driva and Skibotn are the most likely to experience temperatures more resembling the extra low temperature treatment (Fig. 2). Figgjo and Arna did in fact display the largest estimated growth rates of the wild strains in the low temperature treatment, although not significantly higher than the Vosso strain. While in the extra low temperature treatment, Vosso, Driva and Skibotn displayed the lowest growth rates. Thus, no clear indication of local thermal optima adaption in Atlantic salmon, nor contemporary temperature-driven divergence in survival during early development in farmed and wild salmon, was detected in this study.

In addition to results from the present study, other salmonid studies have failed to provide support for the local optima hypothesis [5, 55, 56]. Indications of thermal adaption to low temperatures have however been observed in brown trout *Salmo trutta* populating cold rivers [57]. Also, indications of increased thermal tolerance has been documented in domesticated rainbow trout *Oncorhynchus mykiss*, either as consequence of selective breeding for this trait [58], or due to hitchhiking selection [59]. Heritable variation for thermal tolerance has also been documented in other fish species [31–34].

#### The countergradient hypothesis

Increased growth rates, as an adaption to harsh conditions has been hypothesised for populations inhabiting cold rivers with short growth seasons at high latitudes, i.e., the counter-gradient variation hypothesis [52, 53]. According to this hypothesis, Skibotn in particular, should display higher growth rates than the other wild strains at all temperatures (Figs 1 and 2). This was not the case; in fact, both Skibotn and Driva distinguished themselves by displaying higher mortality rates and smaller growth rates than all other strains, in particular at cold temperatures. Also the Vosso strain displayed a low growth rate in the cold temperature treatments. This result could be influenced by the fact that these strains have been reared in the Norwegian Gene Bank for Atlantic salmon under hatchery conditions for at least part of their life cycle, and thus subjected to warmer temperature conditions during start feeding. However, several studies have investigated counter-gradient thermal adaption without being able to demonstrate this phenomenon in Atlantic salmon [3, 5]. Despite these results, faster growth in populations originating from novel environments have been suggested in other fish species [60].

### Conclusions

Each year, hundreds of thousands of domesticated salmon escape from fish farms into the wild, and many of these migrate onto the spawning grounds of native populations. As a result, genetic changes have been observed in a number of wild populations [29, 38, 61]. Given that farmed strains may display reduced genetic variation in relation to wild salmon populations [62, 63], and the offspring of farmed salmon display reduced survival in the wild when compared with the offspring of wild salmon [23–25], there are international concerns over the genetic integrity of native populations. However, although genomic regions associated with farmed and wild salmon survival in the wild have been recently identified [64], the mechanisms underlying the observed differences in survival still remain more or less completely elusive. Furthermore, while the frequency of farmed escapees and native population density are correlated with inter-population patterns of introgression [29, 38], other ecological factors influencing these patterns remain unidentified. Here, we aimed to address this by investigating whether farmed salmon, which have been subject to domestication selection under elevated temperatures during early development, displayed reduced tolerance of cold-water during this critical phase of the life cycle. However, we found no evidence to support this and we conclude that reduced tolerance for cold water during early development is not the sole contributor to the observed lower survival of farmed offspring in the wild [23–25].

The current study was performed under standard hatchery conditions with unrestricted access to feed and overall high survival rates. Thus, it is possible that potential difference in thermal tolerance between farmed and wild salmon may have been masked by the fact that fish of all origins performed well due to the low competition level. Performing a cold temperature study under conditions resembling the wild environment, e.g., semi-natural conditions with competition for feed, and predation, could therefore be beneficial in terms of understanding how ecological factors in combination with biological factors, such as inter-strain competition, influences introgression levels in cold-water rivers.

### Availability of supporting data

The dataset supporting the conclusions of this article is available in the Dryad Digital Repository [65].

## Additional file

Additional file 1:
**Additional table 1–8.** Statistical output. (DOC 266 kb)
