# Supplementary material for: Thermal plasticity in farmed, wild and hybrid Atlantic salmon during early development: has domestication caused divergence in low temperature tolerance?
Source: BMC Evol Biol. 2016 Feb 16;16:38. doi: 10.1186/s12862-016-0607-2 (PMC4754860; doi:10.1186/s12862-016-0607-2)
Supplement: Additional file 1: — Additional table 1–8. Statistical output. (DOC 266 kb) [file 12862_2016_607_MOESM1_ESM.doc]

**Table A1** Model selection of the random effect of the generalized linear mixed effect model used to investigate survival (model 1)

|  |  |  |  |  |  |  |  |  |  |  |  |  |
| --- | --- | --- | --- | --- | --- | --- | --- | --- | --- | --- | --- | --- |
|  |  | Random effects | | |  |  |  |  |  |  |  |  |
| N | Response | T:r* | S:f | T/S:f | Df | AIC | BIC | logLik | Deviance | Chisq | Chi Df | P |
| 8409 | Survival | **x** | **x** |  | 32 | 4458 | 4683 | -2196.7 | 4393.5 |  |  |  |
|  |  | x | x | x | 36 | 4507 | 4761 | -2217.6 | 4435.2 | 0 | 5 | 1 |
|  |  |  |  |  |  |  |  |  |  |  |  |  |

T:r; replicate nested within treatment (random intercept). S:f; family nested within strain (random intercept). T/S:f; family nested within strain, across treatments (random intercept and slope). Df; degrees of freedom. AIC; Akaike information criterion. BIC; Bayesian information criterion. Loglik; log likelihood. Deviance; Chi.sq; value of the Chi square statistics. Chi.Df; the degrees of freedom for the test. P: P-value. The significant random effect structure is marked in bold.*More than 93% of all dead individuals died prior to the splitting of the control replicate treatment, hence these split replicates were pooled in the survival analysis.

**Table A2** Model selection of the fixed effect of the generalized linear mixed effect model used to investigate survival (model 1)

|  |  |  |  |  |  |  |  |  |
| --- | --- | --- | --- | --- | --- | --- | --- | --- |
|  |  | Fixed effects | | | | |  |  |
| N | Response | T x S | T x E | T | S | E | AIC | ∆ AIC |
| 8409 | Survival | x | x | x | x | x | 4457.5 | 19.4 |
|  |  |  | x | x | x | x | 4440 | 1.9 |
|  |  |  |  | **x** | **x** | **x** | **4438.1** | **0** |
|  |  |  |  |  | x | x | 4440.6 | 2.5 |
|  |  |  |  | x |  | x | 4456 | 17.9 |
|  |  |  |  | x | x |  | 4447.4 | 9.3 |
|  |  |  |  |  |  |  |  |  |

T x S; Treatment by strain interaction. T x E; Treatment by egg size interaction. T; Treatment. S; Strain, E; egg size. AIC; Akaike information criterion. ∆ AIC; difference in AIC value. Models displaying less than 2 AIC in difference were considered equally good, thus by the principle of parsimony the best performing simplest model was selected. The significant fixed effect structure is marked in bold.

**Table A3** Model 1: pair wise comparisons in survival between treatments and strains

|  |  |  |  |  |  |  |
| --- | --- | --- | --- | --- | --- | --- |
| Pair wise comparisons | |  | Value | Df | χ2 | *P* |
| Treatments | Control (12 ° C) | Low (5.6 ° C) | 0.65 | 1 | 4.67 | 0.06 |
|  |  | Extra low (3.9 ° C) | 0.72 | 1 | 11.48 | **0.002** |
|  | Low (5.6 ° C) | Extra low (3.9 ° C) | 0.58 | 1 | 1.52 | 0.22 |
|  |  |  |  |  |  |  |
| Strains | Farm1 | Farm2 | 0.50 | 1 | 0.00 | 1 |
|  |  | Figgjo x Farm1 | 0.45 | 1 | 0.38 | 1 |
|  |  | Farm2 x Vosso | 0.36 | 1 | 2.60 | 1 |
|  |  | Figgjo | 0.38 | 1 | 1.74 | 1 |
|  |  | Vosso | 0.44 | 1 | 0.43 | 1 |
|  |  | Arna | 0.53 | 1 | 0.12 | 1 |
|  |  | Driva | 0.81 | 1 | 17.29 | **0.001** |
|  |  | Skibotn | 0.76 | 1 | 11.87 | **0.014** |
|  | Farm2 | Figgjo x Farm1 | 0.45 | 1 | 0.34 | 1 |
|  |  | Farm2 x Vosso | 0.36 | 1 | 2.50 | 1 |
|  |  | Figgjo | 0.38 | 1 | 1.50 | 1 |
|  |  | Vosso | 0.44 | 1 | 0.39 | 1 |
|  |  | Arna | 0.53 | 1 | 0.10 | 1 |
|  |  | Driva | 0.81 | 1 | 16.48 | **0.001** |
|  |  | Skibotn | 0.76 | 1 | 12.13 | **0.012** |
|  | Figgjo x Farm1 | Farm2 x Vosso | 0.41 | 1 | 0.98 | 1 |
|  |  | Figgjo | 0.44 | 1 | 0.56 | 1 |
|  |  | Vosso | 0.50 | 1 | 0.00 | 1 |
|  |  | Arna | 0.58 | 1 | 0.97 | 1 |
|  |  | Driva | 0.84 | 1 | 21.82 | **<0.001** |
|  |  | Skibotn | 0.80 | 1 | 14.87 | **0.003** |
|  | Farm2 x Vosso | Figgjo | 0.53 | 1 | 0.08 | 1 |
|  |  | Vosso | 0.59 | 1 | 0.93 | 1 |
|  |  | Arna | 0.67 | 1 | 3.63 | 1 |
|  |  | Driva | 0.88 | 1 | 30.52 | **<0.001** |
|  |  | Skibotn | 0.85 | 1 | 24.41 | **<0.001** |
|  | Figgjo | Vosso | 0.56 | 1 | 0.50 | 1 |
|  |  | Arna | 0.64 | 1 | 3.01 | 1 |
|  |  | Driva | 0.87 | 1 | 27.12 | **<0.001** |
|  |  | Skibotn | 0.84 | 1 | 18.83 | 0.0004 |
|  | Vosso | Arna | 0.58 | 1 | 1.04 | 1 |
|  |  | Driva | 0.84 | 1 | 22.42 | **<0.001** |
|  |  | Skibotn | 0.80 | 1 | 15.53 | **0.002** |
|  | Arna | Driva | 0.79 | 1 | 14.27 | **0.004** |
|  |  | Skibotn | 0.74 | 1 | 8.66 | 0.07 |
|  | Driva | Skibotn | 0.43 | 1 | 0.70 | 1 |
|  |  |  |  |  |  |  |

Df; degrees of freedom. χ2; value of the Chi square statistics, P; P-value.

**Table A4**: Model selection of the linear mixed effect model used to investigate growth (model 2)

|  |  |  |  |  |  |  |  |  |  |  |  |
| --- | --- | --- | --- | --- | --- | --- | --- | --- | --- | --- | --- |
|  | Random effects | | | |  | Fixed effects | | | | | |
| Response | Variable | Chi.sq | Chi.Df | P |  | Variable | Sum.sq | NumDf | DenDf | F | P |
| Log Weigt | T:r:t | 12.7 | 1 | **<0.001** |  | T x E | 0.00 | 2 | 45.0 | 0.2 | 0.8 |
|  | T/S:f | 110.8 | 5 | **<0.001** |  | T | 25.53 | 2 | 6.9 | 2158.9 | **<0.001** |
|  |  |  |  |  |  | E | 0.23 | 1 | 140.2 | 39.1 | **<0.001** |
|  |  |  |  |  |  | S | 0.65 | 8 | 27.0 | 13.8 | **<0.001** |
|  |  |  |  |  |  | T x S | 0.63 | 16 | 48.1 | 6.7 | **<0.001** |
|  |  |  |  |  |  |  |  |  |  |  |  |

Significance levels of random and fixed effects included in the full LME models investigating variation in log body weight at termination. Log weight; log10 (wet weight+1) at termination. Random effects: T:r:t, tank (t) nested within replicate (r) and treatment (T) (random intercept). T/S:f, families (f) nested within strain (S), across treatments (T) (random intercept and slope). Chi.sq; value of the Chi square statistics. Chi.Df; the degrees of freedom for the test. P; P-value of the likelihood ratio test for the random effect. Fixed effects: T; Temperature treatment. E; mean family log10 egg size. S, Strain. Sum.sq; sums of squares. Num.Df; numerator degrees of freedom. Den.Df; denominator degrees of freedom (Satterthwaite’s approximation) F; F-value. P; P-value. Significant effects are marked in bold.

**Table A5** Model 2: pair-wise comparisons of growth between treatments

|  |  |  |  |  |  |  |  |
| --- | --- | --- | --- | --- | --- | --- | --- |
| Treatment |  | SE | DF | T | Lower CI | Upper CI | P |
| Control (12 ° C) | Low (5.6 ° C) | 0.02 | 9.6 | 55.7 | 0.96 | 1.04 | **<0.001** |
|  | Extra low (3.9 ° C) | 0.02 | 10.3 | 60.9 | 1.08 | 1.16 | **<0.001** |
| Low (5.6 ° C) | Extra low (3.9 ° C) | 0.02 | 4.1 | 6.9 | 0.07 | 0.16 | **0.002** |
|  |  |  |  |  |  |  |  |

Parameter level tests, i.e., differences of least square means, for the fixed effect of treatment in the final LME model investigating difference in log10 body weight at termination. SE; standard error. Df; degrees of freedom. CI; confidence intervals. Significant results are marked in bold.

**Table A6** Model 2: pair-wise comparisons of growth between strains in the control treatment

|  |  |  |  |  |  |  |  |
| --- | --- | --- | --- | --- | --- | --- | --- |
| Strain |  | SE | DF | T | Lower CI | Upper CI | P |
| Farm1 | Farm2 | 0.0542 | 25.6 | 1.68 | -0.0203 | 0.2027 | 0.11 |
|  | Figgjo x Farm1 | 0.0543 | 25.8 | 3.25 | 0.0646 | 0.2879 | **0.003** |
|  | Farm2 x Vosso | 0.054 | 25.2 | 3.46 | 0.0755 | 0.2978 | **0.002** |
|  | Figgjo | 0.0541 | 25.5 | 6.72 | 0.2522 | 0.4749 | **<0.001** |
|  | Vosso | 0.0542 | 25.6 | 4.42 | 0.1281 | 0.3511 | **<0.001** |
|  | Arna | 0.0544 | 25.9 | 6.15 | 0.2228 | 0.4464 | **<0.001** |
|  | Driva | 0.0603 | 28.7 | 8.01 | 0.3596 | 0.6066 | **<0.001** |
|  | Skibotn | 0.0543 | 25.7 | 6.27 | 0.2289 | 0.4521 | **<0.001** |
| Farm2 | Figgjo x Farm1 | 0.0543 | 25.9 | 1.57 | -0.0266 | 0.1968 | 0.13 |
|  | Farm2 x Vosso | 0.0539 | 25 | 1.77 | -0.0155 | 0.2065 | 0.09 |
|  | Figgjo | 0.0542 | 25.6 | 5.02 | 0.1609 | 0.3839 | **<0.001** |
|  | Vosso | 0.0542 | 25.7 | 2.74 | 0.0369 | 0.26 | **0.01** |
|  | Arna | 0.0544 | 26 | 4.47 | 0.1316 | 0.3553 | **<0.001** |
|  | Driva | 0.0603 | 28.5 | 6.5 | 0.2686 | 0.5153 | **<0.001** |
|  | Skibotn | 0.0541 | 25.4 | 4.61 | 0.138 | 0.3606 | **<0.001** |
| Figgjo x Farm1 | Farm2 x Vosso | 0.0541 | 25.3 | 0.19 | -0.1008 | 0.1217 | 0.85 |
|  | Figgjo | 0.054 | 25.3 | 3.47 | 0.0761 | 0.2985 | **0.002** |
|  | Vosso | 0.0542 | 25.6 | 1.17 | -0.0481 | 0.1749 | 0.25 |
|  | Arna | 0.0543 | 25.9 | 2.92 | 0.0467 | 0.2701 | **0.01** |
|  | Driva | 0.0604 | 28.8 | 5.08 | 0.1833 | 0.4304 | **<0.001** |
|  | Skibotn | 0.0544 | 26.1 | 3.02 | 0.0524 | 0.2761 | **0.01** |
| Farm2 x Vosso | Figgjo | 0.0539 | 25 | 3.28 | 0.0659 | 0.2879 | **0.003** |
|  | Vosso | 0.054 | 25.2 | 0.98 | -0.0581 | 0.164 | 0.34 |
|  | Arna | 0.0541 | 25.5 | 2.73 | 0.0366 | 0.2593 | **0.01** |
|  | Driva | 0.0601 | 28.2 | 4.93 | 0.1734 | 0.4195 | **<0.001** |
|  | Skibotn | 0.0539 | 25.1 | 2.85 | 0.0428 | 0.2649 | **0.01** |
| Figgjo | Vosso | 0.054 | 25.2 | -2.3 | -0.235 | -0.0128 | **0.03** |
|  | Arna | 0.0541 | 25.4 | -0.54 | -0.1402 | 0.0823 | 0.60 |
|  | Driva | 0.0602 | 28.5 | 1.98 | -0.0037 | 0.2428 | 0.06 |
|  | Skibotn | 0.0543 | 25.8 | -0.42 | -0.1348 | 0.0887 | 0.68 |
| Vosso | Arna | 0.0542 | 25.7 | 1.75 | -0.0166 | 0.2065 | 0.09 |
|  | Driva | 0.0603 | 28.7 | 4.04 | 0.1201 | 0.3668 | **<0.001** |
|  | Skibotn | 0.0543 | 25.9 | 1.86 | -0.0108 | 0.2126 | 0.08 |
| Arna | Driva | 0.0604 | 28.9 | 2.46 | 0.0249 | 0.2721 | **0.02** |
|  | Skibotn | 0.0545 | 26.2 | 0.11 | -0.1062 | 0.1179 | 0.92 |
| Driva | Skibotn | 0.0603 | 28.7 | -2.36 | -0.2661 | -0.0191 | **0.03** |
|  |  |  |  |  |  |  |  |

Parameter level tests, i.e., differences of least square means, for the fixed effect of strain in the control treatment in the final LME model investigating difference in log10 body weight at termination. SE; standard error. Df; degrees of freedom. CI; confidence intervals. Significant results are marked in bold.

**Table A7** Model 2: pair-wise comparisons of growth between strains in the low temperature treatment

|  |  |  |  |  |  |  |  |
| --- | --- | --- | --- | --- | --- | --- | --- |
| Strain |  | SE | DF | T | Lower CI | Upper CI | P |
| Farm1 | Farm2 | 0.0186 | 75 | 3.34 | 0.0251 | 0.0992 | **0.001** |
|  | Figgjo x Farm1 | 0.0186 | 75.5 | 1.47 | -0.0096 | 0.0645 | 0.15 |
|  | Farm2 x Vosso | 0.0182 | 70.7 | 2.45 | 0.0082 | 0.0809 | **0.02** |
|  | Figgjo | 0.0184 | 70.7 | 2.4 | 0.0075 | 0.0809 | **0.02** |
|  | Vosso | 0.019 | 81 | 3.57 | 0.03 | 0.1054 | **<0.001** |
|  | Arna | 0.019 | 81.2 | 3.02 | 0.0195 | 0.0952 | **0.003** |
|  | Driva | 0.0202 | 77.3 | 6.42 | 0.0892 | 0.1695 | **<0.001** |
|  | Skibotn | 0.0193 | 85.5 | 5.71 | 0.0718 | 0.1486 | **<0.001** |
| Farm2 | Figgjo x Farm1 | 0.0182 | 66.5 | -1.9 | -0.0711 | 0.0017 | 0.06 |
|  | Farm2 x Vosso | 0.0175 | 60.6 | -1 | -0.0527 | 0.0175 | 0.32 |
|  | Figgjo | 0.0184 | 65.9 | -0.98 | -0.0547 | 0.0187 | 0.33 |
|  | Vosso | 0.0186 | 72.3 | 0.3 | -0.0316 | 0.0426 | 0.77 |
|  | Arna | 0.0187 | 72.8 | -0.26 | -0.0421 | 0.0326 | 0.80 |
|  | Driva | 0.0196 | 68.5 | 3.43 | 0.0282 | 0.1062 | **0.001** |
|  | Skibotn | 0.0184 | 72.9 | 2.61 | 0.0114 | 0.0847 | **0.01** |
| Figgjo x Farm1 | Farm2 x Vosso | 0.0177 | 62.4 | 0.97 | -0.0183 | 0.0526 | 0.34 |
|  | Figgjo | 0.0175 | 60.3 | 0.96 | -0.0182 | 0.0517 | 0.34 |
|  | Vosso | 0.0183 | 71.2 | 2.2 | 0.0038 | 0.0766 | **0.03** |
|  | Arna | 0.0183 | 72 | 1.63 | -0.0066 | 0.0665 | 0.11 |
|  | Driva | 0.0196 | 69.1 | 5.19 | 0.0627 | 0.1411 | **<0.001** |
|  | Skibotn | 0.019 | 76.5 | 4.36 | 0.045 | 0.1205 | **<0.001** |
| Farm2 x Vosso | Figgjo | 0.0176 | 59.5 | -0.02 | -0.0357 | 0.0349 | 0.98 |
|  | Vosso | 0.0181 | 67.5 | 1.28 | -0.013 | 0.0592 | 0.21 |
|  | Arna | 0.0182 | 67.7 | 0.7 | -0.0235 | 0.0491 | 0.48 |
|  | Driva | 0.0193 | 65.3 | 4.4 | 0.0463 | 0.1233 | **<0.001** |
|  | Skibotn | 0.0182 | 70 | 3.6 | 0.0293 | 0.102 | **<0.001** |
| Figgjo | Vosso | 0.0178 | 63.9 | 1.32 | -0.012 | 0.059 | 0.19 |
|  | Arna | 0.0177 | 63.3 | 0.75 | -0.0222 | 0.0486 | 0.46 |
|  | Driva | 0.0196 | 66.7 | 4.35 | 0.0461 | 0.1242 | **<0.001** |
|  | Skibotn | 0.0192 | 77.4 | 3.43 | 0.0277 | 0.1043 | **0.001** |
| Vosso | Arna | 0.0186 | 76.5 | -0.55 | -0.0474 | 0.0268 | 0.58 |
|  | Driva | 0.02 | 74.1 | 3.08 | 0.0218 | 0.1015 | **0.003** |
|  | Skibotn | 0.0193 | 82.9 | 2.2 | 0.004 | 0.081 | **0.03** |
| Arna | Driva | 0.0201 | 75 | 3.58 | 0.0319 | 0.112 | **<0.001** |
|  | Skibotn | 0.0195 | 83.7 | 2.71 | 0.014 | 0.0916 | **0.01** |
| Driva | Skibotn | 0.0202 | 76.8 | -0.95 | -0.0594 | 0.021 | 0.35 |
|  |  |  |  |  |  |  |  |

Parameter level tests, i.e., differences of least square means, for the fixed effect of strain in the low temperature treatment in the final LME model investigating difference in log10 body weight at termination. SE; standard error. Df; degrees of freedom. CI; confidence intervals. Significant results are marked in bold.

**Table A8** Model 2: pair-wise comparisons of growth between strains in the extra low temperature treatment

|  |  |  |  |  |  |  |  |
| --- | --- | --- | --- | --- | --- | --- | --- |
| Strain |  | SE | DF | T | Lower CI | Upper CI | P |
| Farm1 | Farm2 | 0.0177 | 124.6 | 1.46 | -0.0091 | 0.0609 | 0.15 |
|  | Figgjo x Farm1 | 0.0171 | 110.6 | 0.54 | -0.0246 | 0.0432 | 0.59 |
|  | Farm2 x Vosso | 0.0164 | 96.4 | 1.67 | -0.0052 | 0.06 | 0.10 |
|  | Figgjo | 0.0182 | 133.5 | 0.47 | -0.0274 | 0.0445 | 0.64 |
|  | Vosso | 0.0168 | 104.4 | 1.95 | -0.0006 | 0.0662 | 0.05 |
|  | Arna | 0.0178 | 126.2 | 1.22 | -0.0134 | 0.057 | 0.22 |
|  | Driva | 0.0209 | 175 | 3.58 | 0.0336 | 0.1162 | **<0.001** |
|  | Skibotn | 0.0193 | 168.3 | 2.42 | 0.0086 | 0.0849 | **0.02** |
| Farm2 | Figgjo x Farm1 | 0.0172 | 106.2 | -0.97 | -0.0507 | 0.0175 | 0.34 |
|  | Farm2 x Vosso | 0.0161 | 88.9 | 0.09 | -0.0305 | 0.0335 | 0.93 |
|  | Figgjo | 0.0185 | 131.4 | -0.94 | -0.054 | 0.0193 | 0.35 |
|  | Vosso | 0.0169 | 101 | 0.41 | -0.0267 | 0.0405 | 0.69 |
|  | Arna | 0.018 | 122.4 | -0.23 | -0.0397 | 0.0315 | 0.82 |
|  | Driva | 0.0208 | 168.8 | 2.36 | 0.008 | 0.09 | **0.02** |
|  | Skibotn | 0.0188 | 157.8 | 1.11 | -0.0163 | 0.058 | 0.27 |
| Figgjo x Farm1 | Farm2 x Vosso | 0.0157 | 78.6 | 1.15 | -0.0131 | 0.0493 | 0.25 |
|  | Figgjo | 0.017 | 110 | -0.04 | -0.0345 | 0.0331 | 0.97 |
|  | Vosso | 0.0158 | 83.4 | 1.49 | -0.0078 | 0.0549 | 0.14 |
|  | Arna | 0.0167 | 103.9 | 0.75 | -0.0207 | 0.0457 | 0.46 |
|  | Driva | 0.0203 | 153.9 | 3.24 | 0.0256 | 0.1057 | **0.002** |
|  | Skibotn | 0.0189 | 146.6 | 1.98 | 0.0001 | 0.0748 | **0.05** |
| Farm2 x Vosso | Figgjo | 0.017 | 101.1 | -1.11 | -0.0524 | 0.0148 | 0.27 |
|  | Vosso | 0.0154 | 73.4 | 0.35 | -0.0252 | 0.0361 | 0.73 |
|  | Arna | 0.0165 | 93.3 | -0.34 | -0.0383 | 0.0271 | 0.74 |
|  | Driva | 0.0197 | 141.4 | 2.41 | 0.0086 | 0.0865 | **0.02** |
|  | Skibotn | 0.0179 | 129.6 | 1.08 | -0.0161 | 0.0547 | 0.28 |
| Figgjo | Vosso | 0.0167 | 102.6 | 1.45 | -0.0089 | 0.0574 | 0.15 |
|  | Arna | 0.0175 | 122.9 | 0.75 | -0.0215 | 0.0479 | 0.45 |
|  | Driva | 0.0212 | 174.4 | 3.13 | 0.0245 | 0.1082 | **0.002** |
|  | Skibotn | 0.0201 | 171.2 | 1.9 | -0.0015 | 0.0778 | 0.06 |
| Vosso | Arna | 0.0164 | 97 | -0.67 | -0.0436 | 0.0216 | 0.50 |
|  | Driva | 0.02 | 148.1 | 2.1 | 0.0025 | 0.0817 | **0.04** |
|  | Skibotn | 0.0186 | 140.8 | 0.75 | -0.0229 | 0.0507 | 0.46 |
| Arna | Driva | 0.0209 | 168.4 | 2.55 | 0.0119 | 0.0943 | **0.01** |
|  | Skibotn | 0.0196 | 163.1 | 1.27 | -0.0137 | 0.0636 | 0.21 |
| Driva | Skibotn | 0.0222 | 209.3 | -1.27 | -0.0719 | 0.0155 | 0.21 |
|  |  |  |  |  |  |  |  |

Parameter level tests, i.e., differences of least square means, for the fixed effect of strain in the extra low temperature treatment in the final LME model investigating difference in log10(+1) body weight. SE; standard error. Df; degrees of freedom. CI; confidence intervals. Significant results are marked in bold.
